# The rational use of causal inference to guide reinforcement learning strengthens with age

**DOI:** 10.1038/s41539-020-00075-3

**Published:** 2020-10-27

**Authors:** Alexandra O. Cohen, Kate Nussenbaum, Hayley M. Dorfman, Samuel J. Gershman, Catherine A. Hartley

**Affiliations:** 1 Department of Psychology, New York University, New York, NY 10003 USA; 2 Department of Psychology, Harvard University, Cambridge, MA 02138 USA; 3 Center for Brain Science, Harvard University, Cambridge, MA 02138 USA; 4 Center for Neural Science, New York University, New York, NY 1003 USA

**Keywords:** Human behaviour, Decision making

## Abstract

Beliefs about the controllability of positive or negative events in the environment can shape learning throughout the lifespan. Previous research has shown that adults’ learning is modulated by beliefs about the causal structure of the environment such that they update their value estimates to a lesser extent when the outcomes can be attributed to hidden causes. This study examined whether external causes similarly influenced outcome attributions and learning across development. Ninety participants, ages 7 to 25 years, completed a reinforcement learning task in which they chose between two options with fixed reward probabilities. Choices were made in three distinct environments in which different hidden agents occasionally intervened to generate positive, negative, or random outcomes. Participants’ beliefs about hidden-agent intervention aligned with the true probabilities of the positive, negative, or random outcome manipulation in each of the three environments. Computational modeling of the learning data revealed that while the choices made by both adults (ages 18–25) and adolescents (ages 13–17) were best fit by Bayesian reinforcement learning models that incorporate beliefs about hidden-agent intervention, those of children (ages 7–12) were best fit by a one learning rate model that updates value estimates based on choice outcomes alone. Together, these results suggest that while children demonstrate explicit awareness of the causal structure of the task environment, they do not implicitly use beliefs about the causal structure of the environment to guide reinforcement learning in the same manner as adolescents and adults.

## Introduction

The ability to effectively adjust behavior in response to positive and negative feedback is crucial for attaining one’s goals throughout the lifespan. While a growing body of research has aimed to characterize how the ability to learn from feedback changes from childhood to adulthood^1–5^, the vast majority of developmental studies have employed simple task designs in which the probability of obtaining reward depends only on an individual’s own actions^6^. However, many real-world contexts are more complex, with positive and negative outcomes elicited by external causes that are beyond one’s control. For example, the value of an action as simple as carrying an umbrella depends on the likelihood of rain, which may itself depend on unobservable causes. In such cases, a simple learning algorithm that associates actions with reward values will fail to promote optimal behavior. To learn to bring about good outcomes and avoid bad ones, individuals need to infer the extent to which their actions are causally related to the outcomes they experience^7^. Because few studies examining age-related changes in reinforcement learning have manipulated the causal complexity of the learning environment, it is unclear how an individual’s ability to consider their own causal efficacy when learning the value of different actions changes across development.

The extent to which actions and outcomes are causally related indexes the degree of control an individual has over events in that environment. Previous studies in both humans and non-human animals suggest that by adulthood, individuals use their inferences about the controllability of the environment to determine how to adapt their actions to achieve their goals^8^. Critically, for individuals to determine the extent to which adapting their actions in a given context is useful, they must infer how much control they have over positive and negative outcomes in their environment^9^. For example, if a student finds that she does poorly on pop quizzes in a class, she may believe that her bad grades are due to her not studying hard enough. In this case, she might adjust her behavior and study more each night to improve her grades. Alternatively, she may believe that the teacher is a harsh grader and that her grades are due to her teacher’s disposition. In this case, she may not be as likely to update her beliefs about how hard she should work to get a good grade and therefore not adjust her future actions.

The capacity for inferring whether an outcome is due to one’s own actions or due to an external cause can be observed early in development, during infancy^10^. Toddlers are able to infer hidden causes of events and can link hidden causes to both deterministic and probabilistic events^11,12^. Through childhood and adolescence, individuals continue to encounter new, causally complex environments with external sources of good and bad outcomes^13^, such as helpful or harsh teachers. These external causal agents reduce the controllability of the environments in which they operate. However, they often do so systematically, leading to asymmetries in the extent to which positive or negative outcomes are controllable^14^. For example, regardless of an individual’s own actions, a harsh teacher may bring about more negative outcomes, whereas a helpful one may bring about more positive outcomes. It is unclear, however, whether children and adolescents take into account the effects of these external causal agents in assigning credit to their own actions, and in updating their behavior accordingly. Investigating age-related changes in how people understand and use the causal structure of their environments when learning from reinforcement may shed light on how external causes influence adaptive learning and decision-making.

Individuals’ beliefs that external agents asymmetrically influence valenced outcomes should lead to asymmetries in learning from positive and negative outcomes. Specifically, if individuals believe that the outcomes of their actions can be attributed to external causes (like a teacher who grades harshly), and are therefore not under their control, then they should rationally discount the uncontrollable outcomes when assigning credit to their actions. A recent study^15^ tested this hypothesis in adults using a novel reinforcement learning task that included three distinct environments in which hidden agents occasionally intervened to cause positive, negative, or random outcomes. Dorfman et al.^15^ found that participants learned more from positive outcomes in an environment with an adversarial agent that only intervened to generate negative outcomes, and learned more from negative outcomes in an environment with a benevolent agent that only intervened to generate positive outcomes. These results demonstrate that adults adjust the way that they learn from positive and negative outcomes based on their beliefs about the causal structure of their environment. Changes in these beliefs—and the ability to rationally use them to guide value updating—may in part drive age-related differences in learning from similar experiences.

While the ability to understand the properties of causal relationships is evident in early childhood^16^, emerging evidence suggests that learning about causal relations undergoes marked change from childhood, through adolescence, and into adulthood^17–19^. A number of studies conducted in both humans and rodents^19–23^ indicate that adolescents show differences in learning causal relationships in their environment, relative to younger and older individuals. Developmental changes not only in the ability to understand the causal structure of the environment but also to deploy this knowledge in complex environments may lead to age-related differences in learning from reinforcement. Relative to adults, there is emerging evidence that children and adolescents may rely on simpler forms of action-outcome learning that do not incorporate complex knowledge of the reward structure of their environments^2,4^. Thus, while individuals of all ages may be able to demonstrate an understanding of the structure of complex environments, there may be critical developmental changes occurring in the ability to use that understanding to guide performance across childhood and adolescence.

**Fig. 1:**
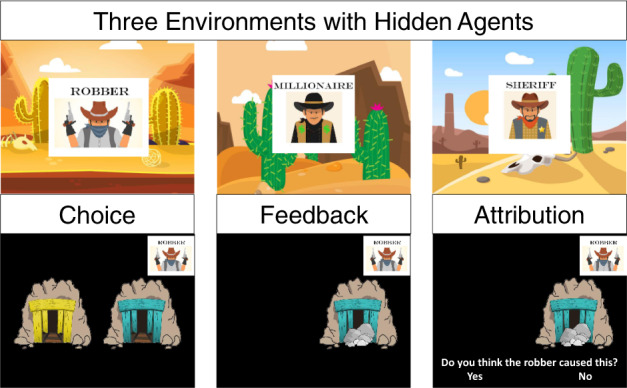
Experimental design. Participants were told that they were mining for gold in three territories visited by three different agents who intervened to generate negative (Robber), positive (Millionaire), or random (Sheriff) outcomes. On each trial, participants selected a mine (Choice), observed the outcome associated with their choice (Feedback), and indicated their belief about hidden agent intervention (Attribution). Elements of this image were designed by Freepik (https://www.freepik.com/) and are licensed for personal use.

In this study, our aim was to determine whether individuals at different ages can (1) infer the latent causes of uncontrollable positive and negative outcomes and (2) incorporate this causal knowledge into their evaluation of the efficacy of their own actions. To address these questions, we leveraged the paradigm developed by Dorfman et al.^15^ and tested 90 individuals ages 7 to 25 years old on a modified version of the task (Fig. 1). Participants were told that they were mining for gold in the Wild West and should try to find as much gold as possible by choosing to dig at the better of two mines. Critically, each block took place within a different territory, each frequented by a different hidden agent. Participants were told a nice millionaire sometimes put gold in both mines, a mean robber sometimes replaced the gold in both mines with rocks, and a sneaky sheriff sometimes randomly put rocks and gold in either mine. After viewing the outcome of each choice, participants had to indicate whether they believed it was caused by the hidden agent. Participants were told the territory they were in, but had no way of knowing if a hidden agent intervened on any given trial.

We first examined causal attribution and learning data separately, and then used computational modeling to assess the influence of the structure of the environment on learning. Informed by previous work across species suggesting age-related changes in both the acquisition of causal structure knowledge and learning from valenced feedback, we hypothesized that beliefs about external causes may be used to guide learning to a greater extent in older than in younger individuals.

## Results

### Behavioral analyses

**Fig. 2:**
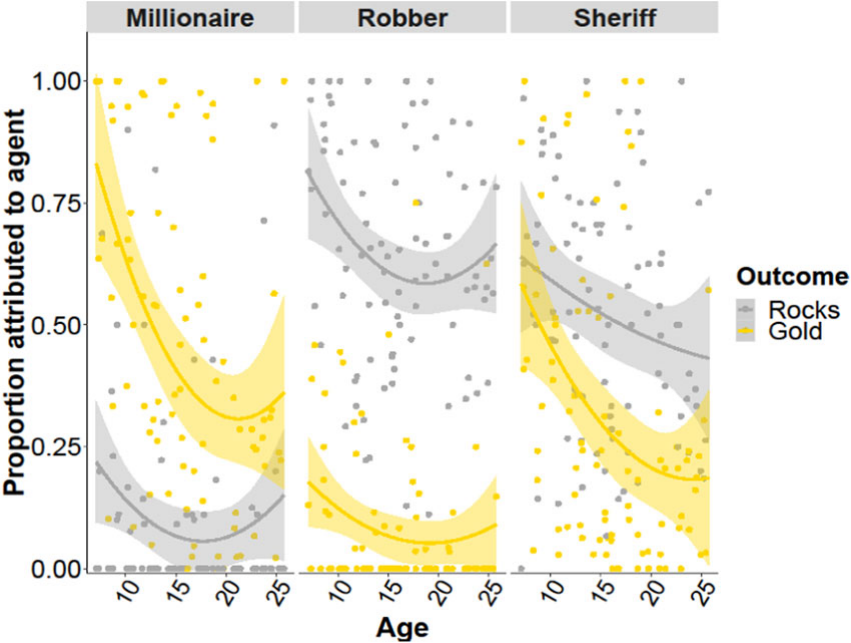
Attribution data. Participant’s beliefs about hidden agent intervention aligned with the experimental manipulation, and attribution rates were higher overall in younger individuals. We also observed an age by reward outcome interaction, indicating that younger individuals attributed positive outcomes to the hidden agent more so than older individuals.

We first examined whether participants’ beliefs about hidden agent intervention aligned with the causal structure of each territory across participants, as a function of age. In other words, we examined trial-wise attributions to the hidden agent by territory (Millionaire, Robber, and Sheriff), reward outcome (gold or rocks), continuous age, continuous age-squared, and their interactions using logistic mixed-effects models. Consistent with the experimental manipulation, there was a significant reward outcome by territory interaction (*χ*^2^(2, *N* = 90) = 87.69, *p* < 0.0001) indicating that participants attributed negative outcomes most often to the Robber, to a lesser extent to the Sheriff, and rarely to the Millionaire while attributing positive outcomes most often to the Millionaire, to a lesser extent to the Sheriff, and rarely to the Robber (Fig. 2). There was also a significant reward outcome by age interaction (*χ*^2^(1, *N* = 90) = 4.85, *p* = 0.028), such that younger individuals tended to attribute positive outcomes to external causes more than older individuals while individuals across the age range were relatively equally likely to attribute negative outcomes to the hidden agents. Further, we observed main effects of territory (*χ*^2^(2, *N* = 90) = 45.04, *p* < 0.0001), reward outcome (*χ*^2^(1, *N* = 90) = 7.44, *p* = 0.006), continuous age (*χ*^2^(1, *N* = 90) = 17.09, *p* < 0.0001), and continuous age squared (*χ*^2^(1, *N* = 90) = 6.14, *p* = 0.013). These effects indicated that participants attributed more outcomes to the hidden agent when in the robber and sheriff conditions and when they received rocks. Younger participants also tended to report that more outcomes were attributable to hidden agents overall. There were no other significant interactions of reward outcome by age-squared, territory by age, territory by age-squared, or three-way interactions (all *χ*^2^ s < 4.0, *p*s > 0.13).

**Fig. 3:**
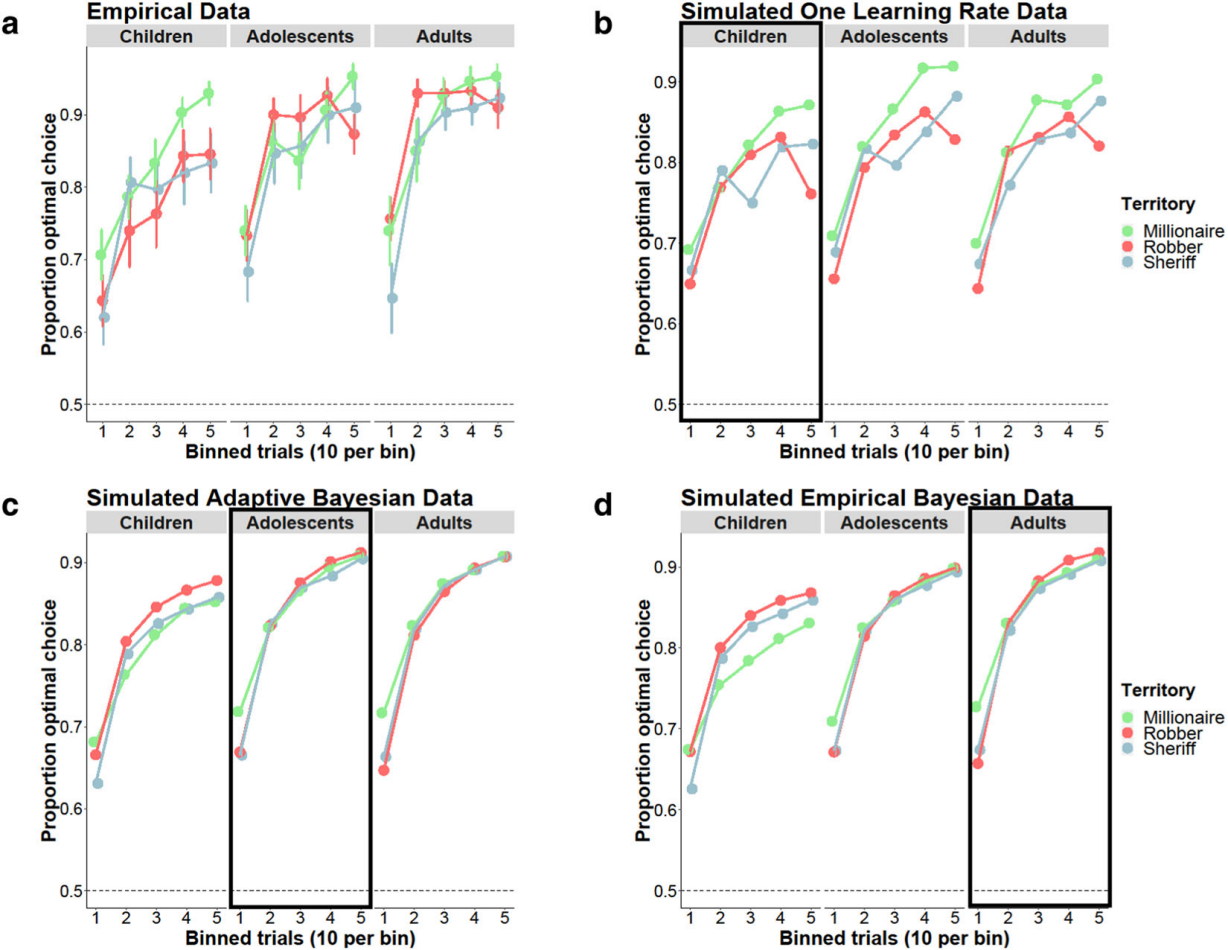
Observed and simulated learning data. Across territories, older participants learned to select the best mine faster. Younger participants showed better learning in the Millionaire territory (**a**). Simulated data using subjects’ fitted parameter estimates for each of the best fitting models are depicted (**b–d**). Boxes denote the age group that was best fit by the model. Participants are separated by age group (Children: 7–12, Adolescents: 13–17, Adult: 18–25) and trial bins for visualization purposes. The corresponding statistical analyses on the empirical data treat age and trial as continuous variables.

We next assessed age-related change in learning across the three distinct environments (Fig. 3a) by examining trial-wise optimal choice by territory, trial number within a territory, continuous age, continuous age-squared and their interactions using logistic mixed-effects models. We found significant main effects of trial number (*χ*^2^(1, *N* = 90) = 100.46, *p* < 0.0001), age (*χ*^2^(1, *N* = 90) = 13.97, *p* < 0.001), and age squared (*χ*^2^(1, *N* = 90) = 5.17, *p* = 0.023), indicating that participants learned to select the more highly rewarded mine more frequently as each block progressed and that older participants selected the better mine on a higher proportion of trials. These main effects were qualified by interactions. There was a significant trial number by territory interaction (*χ*^2^(2, *N* = 90) = 15.40, *p* < 0.001), such that learning trajectories were steeper for the Millionaire and Sheriff territories than the Robber territory. There were also significant territory by age squared (*χ*^2^(2, *N* = 90) = 6.89, *p* = 0.032) and trial number by territory by age-squared (*χ*^2^(2, *N* = 90) = 6.81, *p* = 0.033) interactions, as well as a marginal trial number by age interaction (*χ*^2^(1, *N* = 90) = 3.82, *p* = 0.051). There were no statistically significant effects of territory, territory by age, trial number by age squared, or trial number by territory by age (all *χ*^2^ s < 3.2, *p*s > 0.15). Together, these results suggest that older participants, relative to younger participants, learned faster across all territories and that younger participants showed better learning in the environment where the agent intervened to generate positive outcomes, relative to the other environments.

### Computational modeling

Our central question of interest was whether learning from positive and negative outcomes was differentially influenced by the causal structure of the environment across age groups. To address this question, we fit a set of computational models to participant choice data to determine the model that best captured the learning process for each age group. We fit three reinforcement learning models that did not take into account participant beliefs about hidden-agent intervention when updating the value estimates of choices (one learning rate, two learning rate, three learning rate) and we fit four variants of a Bayesian reinforcement learning model introduced in Dorfman et al.^15^ that incorporated this causal knowledge (empirical Bayesian by territory, adaptive Bayesian, noisy Bayesian, and empirical Bayesian; see “Methods” section for descriptions of each model).

**Fig. 4:**
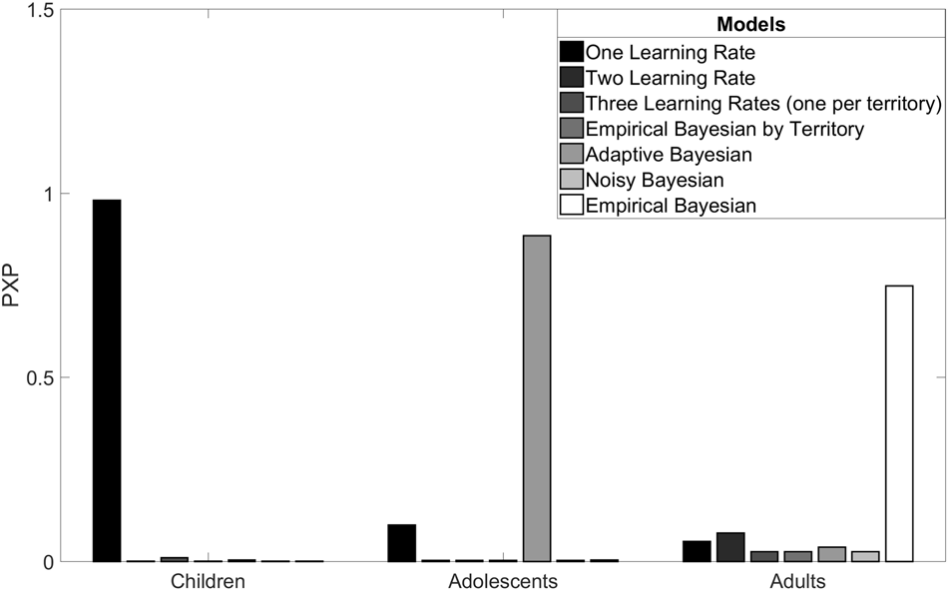
Computational model comparison. Children were best fit by a one learning rate model, adolescents were best fit by the adaptive Bayesian model, and adults were best fit by the empirical Bayesian model, as indexed by protected exceedance probabilities (PXPs).

We compared model fits for one learning rate, two learning rate, three learning rate, empirical Bayesian by territory, adaptive Bayesian, noisy Bayesian and empirical Bayesian reinforcement learning models within three age groups in order to test for age-related differences in the way beliefs about the causal structure of the environment influenced learning. We examined protected exceedance probabilities^24^ (PXPs) for the seven models within each age group (Fig. 4; see Supplementary Figs. 4 and 5 for preferred model frequencies by continuous age). Consistent with the results reported in Dorfman et al.^15^, we found that adult choices were better captured by the empirical Bayesian model (PXP = 0.75) over the other models (all PXPs < 0.08), suggesting that adults showed greater learning from positive outcomes when the agent intervened to produce negative outcomes and greater learning from negative outcomes when the agent intervened to produce positive outcomes. Adolescents were best fit by the adaptive Bayesian model (PXP = 0.89) relative to the other models (all PXPs < 0.10), indicating that their learning was also guided by the structure of their environment but in a more flexible manner, that was less closely tied to their explicitly reported beliefs about latent agent intervention as compared adults. In contrast, children were best fit by the one learning rate model (PXP = 0.98) relative to the other models (all PXPs < 0.01; see Supplementary Table 4 for complete reporting of PXP values). These results indicate that children updated the value of their choices based on experienced outcomes alone and that while they explicitly understood the different structures of the environments, they did not rationally discount outcomes that they could attribute to hidden agents when estimating the value of their actions.

### Model recovery

In order to determine the recoverability of the best-fitting models, we simulated 10,000 participants from each of our models of interest using randomly selected parameters from the empirical distribution of parameter estimates and the empirical distribution of participants’ average attribution judgments (see Table 1 for the mean parameter estimates from the best-fitting models by age group). Trial order for each simulated participant was determined by randomly selecting one of the six possible trial orders and choices were determined via a weighted coin flip. After filtering for accuracy, (greater than 60% optimal choice), 9691 simulated participants remained for the one learning rate model, 9748 simulated participants remained for the adaptive Bayesian model, and 9692 simulated participants remained for the empirical Bayesian model. All models were recoverable (PXP = 1 for all three models).

### Simulations

In order to qualitatively evaluate model fit to the data, we conducted 100 simulations using each subject’s fitted parameters and trial order, resulting in 3000 simulated subjects per age group for each of our best fitting models (Fig. 3b–d). Choices were determined via a weighted coin flip. Relative to the Bayesian models, the one learning rate model shows learning trajectories that differ more across territories (Fig. 3b). This is expected because, once the participant has converged on selecting the optimal mine in all three territories, the participant is least likely to experience large negative prediction errors in the Millionaire territory, where agent intervention always results in reward. In the Robber territory, once the participant has converged on the optimal mine, the participant will occasionally experience large negative prediction errors due to hidden agent intervention, which will cause her to lower her value estimate for the better mine, resulting in the dip in performance observed in the one learning rate model. Qualitatively comparing the simulated results for these models to the empirical learning data for children, adolescents, and adults, we find that the one learning rate simulation mirrors children’s better learning in the millionaire condition, while the Bayesian models reflect the relatively similar learning trajectories across territories demonstrated by adolescents and adults.

## Discussion

The present study examined how manipulating the latent cause of positive and negative outcomes in the environment influences reinforcement learning from childhood to adulthood. We found evidence for age-related differences in beliefs about the causal sources of unpredictable outcomes and—central to this study—in the *use* of causal attributions to guide value-based learning. We found that while children showed generally higher rates of attribution, across age, participants were more likely to attribute positive outcomes to a hidden agent when the agent was benevolent and negative outcomes to the hidden agent when the agent was adversarial, in line with the task structure. However, our computational modeling results suggest that while most adolescents and adults took these attributions into account when estimating the value of their actions in each environment, most children did not do so in a similar manner. These findings add to the growing literature examining reinforcement learning across development^3,5,25–27^ and suggest that the evaluation of actions may become increasingly sensitive to beliefs about the causal structure of the environment with age.

We found that participants of all ages made causal attributions that aligned with the true intervention structure of the task. This finding is consistent with studies showing that the ability to understand causal relationships and make causal inferences is evident during early childhood^16,28–30^. Our causal attribution data also revealed that younger participants were more likely to attribute positive versus negative outcomes to hidden agents relative to older participants. This result in children contrasts with findings of an optimistic bias in adults, such that adults have the tendency to attribute bad outcomes to an external cause more so than good outcomes^15,31^. Increased optimistic biases have been associated with greater perceived control over the environment^32,33^. Thus, it is possible that younger participants, who show higher rates of attribution overall and who likely have less control over events in their day-to-day lives, might demonstrate this bias to a lesser extent. Perceived control over life events can dramatically shape behavioral responses, which can ultimately confer risk or resilience to psychopathology^34,35^. Further research is necessary to better understand the typical development of cognitive mechanisms underlying the formation of beliefs about external causes in order to delineate windows during which perceived control may have particularly lasting effects on behavior.

To address our primary question of interest, we assessed age-related differences in the ability to *use* beliefs about the causal structure of the environment to guide reinforcement learning. We found that while most adolescents and adults were best fit by Bayesian models, which incorporated the structure of the environment into learning, most children were best fit by a simpler one learning rate model that only took into account their choices and the resulting outcomes. These findings align with previous work suggesting that children may rely more on simple stimulus-action associations to guide value-based decision making rather than on their internal model of the structure of the environment^2^. Decker et al.^2^ found that more complex model-based strategy use began to emerge in adolescence and became stronger in adulthood, but was not evident in children, who used a model-free, habitual learning strategy that relies on repetition of previously rewarding actions. However, in line with the present study, there were no significant differences across age in knowledge of task structure as indexed by both explicit and implicit measures.

This study also suggests a subtle difference in how adolescents and adults incorporate information about the structure of the environment into learning. The non-linear age-related change in causal attributions was driven by adolescents’ lower rates of attributing outcomes to the external agents across all environments. This result suggests that adolescents may have an elevated belief in their own control over their environment and is consistent with emerging evidence that adolescents show increased confidence in their decision making^36^ and tend to underweight rare outcomes^37^, like the occasional intervention of a hidden agent. In addition, our computational modeling results suggest that adolescents also demonstrated more flexibility in how they learned from feedback, a result consistent with recent findings^38^. Adolescents were best fit by the adaptive Bayesian model, which estimated the intervention probability on each trial, whereas adults were best fit by the empirical Bayesian model, which incorporates participant’s own attribution judgments into the model, rather than allowing the model to estimate the intervention probability. This result suggests that adolescents’ learning was less related to their reported beliefs about latent agent intervention as compared adults, who tend to exhibit more optimistic causal attribution biases. In other words, adolescents tended to flexibly update their beliefs about the structure of the environment throughout the task, and used that changing representation to guide their choices.

The ability to make choices based on flexible representations of the environment that update dynamically with new experiences may be particularly important during adolescence. In the real world, adolescents are often faced with new opportunities for making choices across varied environments^39^. This shift in autonomous experiences coincides with improvement in cognitive domains that are key for making decisions in complex contexts. For example, age-related increases in flexibly incorporating information to solve problems (fluid reasoning) have been shown to mediate the relationship between age and the use of a model-based learning strategy^40^. In the absence of extensive prior experience in various contexts, it may be advantageous for adolescents to rely on a more flexible learning strategy that simultaneously estimates the value of different actions *and* key properties of the causal context itself. Although it is difficult to pinpoint precisely when a cognitive system becomes functionally mature, the present study, together with previous findings^2^, indicates that the ability to reliably use mental models of the environment to guide learning may strengthen during adolescence. Our results suggest that adolescence may represent an important period during which individuals move away from decisions driven by recently experienced rewards^41,42^ toward more deliberative incorporation of mental models of environments during learning.

The emerging use of more complex learning strategies during adolescence may be due to developmental changes in the neural systems that support more complex, model-based learning strategies^2,39^. While the precise neural mechanisms underlying the learning processes in the current task have yet to be elucidated, prefrontal-hippocampal-striatal circuitry has been implicated in the use of mental models of the environment to guide learning^43–45^. In addition, communication between medial prefrontal cortex and subcortical brain areas is proposed to be critical for expressing proactive behavioral responses associated with higher estimates of control in the environment^8^. Both prefrontal cortical and hippocampal systems show protracted development into and across adolescence^46–49^ which may contribute to the observed age-related changes in using inferred latent causes to assign credit to actions.

Consistent with previous studies that have examined probabilistic reinforcement learning from childhood to adulthood^1,4^, we found that older participants outperformed younger participants across all learning contexts. We found that younger participants showed better learning in an environment where a hidden agent occasionally intervened to generate positive outcomes relative to the other learning environments. In other words, younger participants showed better learning in a context in which negative outcomes were most informative, as negative outcomes could only be attributed to the choice the participant made and not to a hidden agent. This result aligns with earlier work showing that children tend to update their value estimates more in response to recent negative outcomes relative to recent positive outcomes^5,50^. Still, several studies have also found no differences in learning for positive and negative outcomes^3,51^ which suggests that more work is needed to understand the contexts in which positive or negative outcomes may be more heavily weighted in learning processes across development.

The current findings highlight several additional avenues for further research. While simulations of the best fitting models show qualitatively similar patterns to the learning results reported here, we still find heterogeneity in the best fitting models within each age group, particularly in younger participants (see Supplementary Figs. 4 to 6). Although younger participants’ choices, on the group level, are best described by the one learning rate model, it is possible that younger participants incorporated their beliefs about the causal structure of the environment into learning in a manner that is not captured by the Bayesian learning models implemented here. For example, children, relative to older individuals, may have different priors on the likelihood that good or bad outcomes can be attributed to external causes. Consistent with prior work^19,52,53^, we also observed increased variability in children’s choice behavior as compared to adults, which may have important consequences for understanding developmental differences in learning mechanisms. Studies of reinforcement learning across development have not yet demonstrated consistent age-related changes in how individuals weight different outcomes when updating the estimated value of different actions^6^. The lack of convergence across studies suggest that more carefully accounting for and manipulating task structure, or the context in which learning takes place, may be critical for understanding age-related differences in reinforcement learning processes. Future studies that build and test hypothesis driven models of how children and adolescents incorporate beliefs about the structure of the environment into reinforcement learning will help us gain a more nuanced understanding of the developmental differences in learning from positive and negative outcomes in various contexts.

In addition, in our task, the influence of external causes was both invisible and ambiguous. Although participants always knew whether the hidden agent could cause positive, negative, or both types of outcomes, they had no way of knowing whether the outcome of any specific choice was due to their action or an agent’s intervention. Thus, they had to rely on their own causal inference to assign credit to potential sources of the outcomes. Though children attributed outcomes to hidden agents *more* than adolescents and adults, they may have been less certain about these attributions, and therefore ignored them, or relied on them to a lesser extent when assigning credit to their actions. While previous work has suggested that young children, and even infants, can infer the causal efficacy of hidden sources^54,55^, it is unclear how their confidence in these inferences—and subsequent use of them—compares to their understanding of the effects of observable causal agents. Future experiments using observable agent interventions could directly test whether children fail to use more explicit causal information to guide value-based learning or whether the effects such as those reported here are specific to the case when they must rely on their own inference about hidden causes.

The present results also provide preliminary insights into distinctions between the understanding and use of causal knowledge. Our results demonstrate that individuals can learn about and gain an explicit understanding of the causal structure of their environments but fail to use that knowledge to guide action selection. This suggests an asymmetry in the relation between causal knowledge and reinforcement learning: using action-outcome associations to learn the causal structure of the environment likely relies on a different learning mechanism than using causal knowledge to learn the optimal action selection policies. Indeed, emerging evidence from computer science research suggests that model-free meta-reinforcement learning can give rise to generalizable causal reasoning^56^. We suggest that the use of causal knowledge for action selection in complex, probabilistic environments may require different learning processes than this acquisition of causal understanding. Future work focusing on the degree of overlap between these learning mechanisms will further contribute to our understanding of how mental models guide learning.

Across the lifespan, individuals encounter many scenarios in which hidden, external causes trigger positive or negative outcomes. For example, even after eating healthfully, people may get sick from unobservable germs; even after acting with kindness, kids sometimes get snapped at by parents who are having a bad day; even after diligently studying, students can perform poorly on exams graded by a harsh teacher. Appropriately discounting the influence of these causes—the hidden germs, the parent’s bad day, the harshness of the teacher—is critical when learning to estimate the value of one’s own actions. The present study replicates and extends previous work in adults examining the influence of beliefs about the causal structure of the environment on learning from positive and negative outcomes. Our results indicate that while children, adolescents, and adults demonstrate an understanding of different causal structures within the task, with increasing age, individuals begin to incorporate inferences about the controllability of external causes when assigning credit to their actions. This work examining how the environment influences learning from childhood to adulthood helps shed light on observed developmental changes in reinforcement learning and highlights several future lines of inquiry at the intersection of developmental and computational cognitive science.

## Methods

### Participants

Ninety participants between the ages of 7 and 25 years-old (M_age _= 15.89, SD_age_ = 5.24, 47 female) were included in analyses. A target sample size of *n* = 90, including 30 children, 30 adolescents, and 30 adults, was determined prior to data collection based on similar studies of learning across development that performed model comparison across age groups^1,4,27,57^. We included children as young as age 7 due to task piloting that indicated that this was the youngest age at which children reliably understood the task instructions. In the present study, children ranged in age from 7–12 years (M_age_ = 10.13, SD_age_ = 1.89), adolescents ranged in age from 13–17 years (M_age _= 15.54, SD_age_ = 1.50), and adults ranged in age from 18–25 years (M_age_ = 21.99, SD_age_ = 2.34). Age bins were based on prior work, which often considers adolescents as individuals aged 13 to 17 years^2,3,5,27^. Data from 12 additional participants (age range: 7–24 years, M_age_ = 13.64, SD_age_ = 6.33, 4 female) were excluded from all analyses for failing to meet the performance criteria of selecting the better choice option (see “Reinforcement learning task” section) on more than 60% of trials^15^. Participants comprised a sample of volunteers recruited from the local community of New York City. Of the 90 participants included in the analyses, 46.67% identified as Caucasian/White, 11.11% as African American, 27.78% as Asian, 0.01% as Native American, and 13.33% as mixed race. In addition, 16.67% of the sample identified as Hispanic. Based on self-report or parental-report, all participants had no history of psychiatric diagnoses, learning disabilities, use of beta blockers or psychoactive medications, or colorblindness. Adult participants provided informed written consent and minor participants provided assent, according to research procedures approved by New York University’s Institutional Review Board. Parents or guardians of participants under age 18 also provided written consent on behalf of the child prior to participation in the study. The research took place during a single session and all participants were compensated $15 per hour plus a $5 bonus.

### Reinforcement learning task

Participants completed a version of the reinforcement learning task introduced in Dorfman et al.^15^, which we adapted for use in developmental populations (Fig. 1). Participants were told that they were mining for gold in the Wild West and that they would earn a small amount of real bonus money each time they found gold and lose a small amount of real bonus money each time they found rocks. On each trial, participants were presented with two differently colored mines, and had to select one at which to dig for gold by pressing its corresponding button on a standard keyboard (Fig. 1). After making each selection, participants were presented with either gold or rocks in front of the selected mine for 2 s. Within each block, one mine produced gold with an 80% probability while the other mine produced gold with a 20% probability. The mines remained on the same sides of the screen for the duration of the block. Participants were told that within each block, they should try to discover and continue to select the mine they believed was more likely to provide gold.

Participants completed three blocks of 50 trials each. Critically, each block took place within a different territory, in which a different hidden agent intervened on the mines on 30% of trials. Participants were instructed about each of the hidden agents prior to beginning the task. Participants were told that in millionaire territory, a nice millionaire sometimes put gold in both mines, such that the participant would receive gold regardless of which mine they selected. In robber territory, a mean robber sometimes replaced the gold in both mines with rocks, such that the participant would receive rocks regardless of their choice. And in sheriff territory, a sneaky sheriff sometimes randomly put rocks and gold in either mine. Participants were told that the hidden agents intervened “on a small number of trials,” but they were not told the exact intervention probability, which was fixed at 30% in each territory. This meant that, on average, in millionaire territory, the better mine yielded gold on 85.74% of trials while the worse mine produced gold with 42.65% probability. In robber territory, on average, the better mine yielded gold on 55.62% of trials and the worse one on 10.89% of trials. And in sheriff territory, on average, the better mine yielded gold on 71.15% and the worse on 29.37%. Prior to beginning each block, participants were told which territory they were in and a picture of the hidden agent remained visible on the corner of the screen for the duration of the trials within that territory. After viewing the outcome of each choice (feedback) for two seconds, participants had to indicate whether they believed it was caused by the hidden agent with a “yes” or “no” response. Selection of the mine (choice) and indication of belief about hidden agent intervention (attribution) were both self-paced. Importantly, they had no way of knowing with certainty whether or not the agent intervened on each trial.

Prior to beginning the real trials, participants first completed five practice trials consisting of directed choices between two mines in order to demonstrate the probabilistic nature of the choice outcomes. Next, participants completed five practice trials in each territory. During the practice trials, to ensure that participants understood the task, an experimenter corrected participants if they ever indicated that an agent made an impossible intervention. For example, if a participant received rocks in millionaire territory and then indicated that they believed the millionaire had caused this outcome, an experimenter would say,” Remember, the millionaire only leaves gold in the mines.” Experimenters corrected participant responses only during these practice trials, and not throughout the experimental trials included in the analyses. Six different versions of the task were selected from 50 randomly generated trial orders in order to ensure that different versions maintained similar reward probabilities and differences between reward probabilities across and within blocks (territories), once interventions were taken into account. Territory order was counterbalanced across participants. The task was programmed in PsychoPy Version 1.85.6^58^.

### Analysis approach

Data processing and statistical analyses were conducted in R version 3.5.1^59^. Logistic mixed-effects models were run using the “lme4” package (version 1.1–17) glmer function^60^ for trial-wise analyses of beliefs about hidden causes (attributions) and learning. We used the maximal model^61^ including a single random intercept per participant and random slopes for within-subjects fixed effects and their interactions. Statistical significance of the fixed effects is reported from analysis of the deviance (Type III Wald chi-square tests) performed on the maximal models for attribution and learning. Age was treated as a continuous variable in these analyses. Numeric variables included as regressors in the model (age and trial number) were z-scored across all participants. We fit models using a mean-centered linear age predictor and a squared mean-centered age term in order to test for non-linear effects of age^62^ and we compared models by likelihood ratio chi-square test to select the best fitting model. For both analyses, models including an age-squared term—along with the linear age term—fit best (attributions: *χ*^2^(6) = 42.43, *p* < 0.0001, learning: *χ*^2^(6) = 39.85, *p* < 0.0001). All reinforcement learning model analyses were completed in MATLAB R2016a.

### One, two, and three learning rate models

The one learning rate model—a standard temporal difference model—assumes that the extent to which participants update their beliefs about the value of the mines ($\theta$) is based only on whether their experienced outcome (*r*) is better or worse than they expected, such that:

1 $$\theta _{t + 1} = \theta _t + \alpha _t\left( {r_t - \theta _t} \right)$$

The two learning rate model is distinguished by having separate learning rates for positive and negative prediction errors, where $\alpha = \alpha _ + \,if\,\left( {r_t - \theta _t} \right)\,>\,0$ and $\alpha = \alpha _ - \,if\,\left( {r_t - \theta _t} \right)\,<\,0$. Critically, these models assume that the learning rates are insensitive to the causal structure of the environment, and therefore are consistent across all three experimental blocks. Both the two learning rate and the Bayesian reinforcement learning models described below assume that there are valence-dependent learning asymmetries within each environment; therefore, we included a one learning rate model that does not have this built-in assumption.

The three learning rates model differs from the one and two learning rate models in that there is a separate learning rate ($\alpha$) for each territory. The separate learning rates allow for differences between territories in the weighting of recent experienced outcomes when updating the value of the mines.

### Empirical, empirical by territory, adaptive, and noisy Bayesian models

The empirical Bayesian model^15^ assumes that participants take into account the possibility that an experienced outcome was caused by a hidden agent when updating their estimates for the value of each mine. As with the one and two learning rate models, after choosing a mine and experiencing a reward (*r*), participants update their estimate of the value of the mine ($\theta$) by multiplying the prediction error they experienced by their learning rate ($\alpha$).

Critically, here the learning rate is dynamically modulated by the posterior probability that an outcome was caused by a hidden agent on each trial, such that participants update their value estimates to a lesser extent when they believe a trial’s outcome can be attributed to the agent. On each trial, the posterior probability of agent intervention is computed by taking into account the probability that a given mine would have led to the experienced outcome with and without an agent intervention, as well as the participant’s prior belief in the probability of an agent intervening. Here, we assume that each participant has a different estimate of the prior probability of agent intervention, which we derive by computing the proportion of trials across the experiment in which they indicated that they believed outcomes were caused by hidden agents.

Participants’ learning rates are then scaled by the posterior probability of agent intervention on each trial. For example, if a participant receives rocks in the Robber territory, the learning rate will be reduced proportionally to the participant’s belief that the hidden agent was responsible for the outcome. However, if a participant receives gold in the Robber territory, it is not possible that this outcome was due to the Robber and the update rule is equivalent to a standard reinforcement learning update rule (see Supplementary Information for full mathematical description). The model thus implements a value update policy such that participants with high rates of agent attributions will demonstrate large asymmetries in the weights they place on positive and negative outcomes across territories. A participant who thinks the agent intervenes often will weigh positive outcomes in robber territory much more heavily than negative outcomes, which she will rationally discount. This same participant will demonstrate the opposite learning bias in millionaire territory, in which she will discount positive outcomes and more heavily weigh negative outcomes.

The empirical Bayesian by territory model differs from the empirical Bayesian model in that it incorporates different estimates of the prior probability of agent intervention. Intervention probabilities are derived by computing the proportion of trials within a territory and for a given outcome (e.g., rocks in the Robber territory) in which the participant indicated that they believed the outcomes were caused by a hidden agent. The adaptive Bayesian model^15^ (see Supplementary Information for mathematical description) does not use empirically derived probabilities of agent intervention, but instead estimates the intervention probability on each trial from experience. In other words, participants learn the overall probability of agent intervention over the course of the task. Finally, the noisy Bayesian model is a variant of the empirical Bayesian model that incorporates an “intervention variability” parameter epsilon, to allow noise in the inferred intervention probabilities. The three other Bayesian models assume that participants only believe that possible interventions occur (e.g., they only believe the millionaire intervened on trials in which they received gold). The noisy Bayesian model instead assumes that participants may sometimes believe in impossible interventions (e.g., they believe the millionaire intervened on trials in which they received rocks). We assume that if participants think an intervention was made, they believe the hidden agent intervened to cause an impossible outcome with probability epsilon and a possible outcome with probability 1-epsilon. Thus, if epsilon is 0, the model reduces to the original empirical Bayesian model in which participants only believe in possible interventions.

### Choice function

For all three reinforcement learning models, we assume that participants’ value estimates probabilistically influence their choices^63^. We implement this by inputting the estimated values of the mines into a softmax choice function to model choice probabilities, with an inverse temperature ($\beta$) parameter and a“stickiness” parameter ($\emptyset$) to capture each individual’s tendency to repeat or switch choices, such that the probability of selecting mine 1 is:

2 $$\frac{{e^{\beta \ast \theta _1 + \emptyset \ast I_1}}}{{e^{\beta \ast \theta _1 + \emptyset \ast I_1} + e^{\beta \ast \theta _2 + \emptyset \ast I_2}}}$$

where *I* is 1 if the mine was selected on the previous trial, and 0 otherwise.

### Model comparison

As in Dorfman et al.^15^, we used random-effects Bayesian model selection to compare model fits using mfit (https://github.com/sjgershm/mfit), and the Laplace approximation of the log marginal likelihood to obtain model evidence values. This procedure assumes that each participant is drawn from a single population, with some distribution over models. Because we were interested in whether age systematically influenced the underlying learning and choice mechanisms that our models of interest may approximate, we binned our participants into three age groups: children (ages 7–12), adolescents (ages 13–17), and adults (ages 18–25), which allowed for the possibility that each group population might be characterized by a different model distribution. We computed the protected exceedance probability (PXP) separately for each model within each age group. The PXP indicates the probability that a model is more frequent than the other models within a comparison set, over and above chance^24^, for the participants included in the group.

**Table 1:** Estimated parameter means (and standard errors).

	One learning rate			Adaptive Bayesian		Empirical Bayesian	
	α	*β*	*ϕ*	*β*	*ϕ*	*β*	*ϕ*
Children	**0.38 (0.04)**	**4.85 (0.30)**	**0.38 (0.17)**	3.80 (0.29)	0.52 (0.15)	3.90 (0.31)	0.57 (0.14)
Adolescents	0.35 (0.04)	5.72 (0.48)	1.02 (0.19)	**4.87 (0.39)**	**0.94 (0.15)**	4.71 (0.37)	1.02 (0.16)
Adults	0.33 (0.04)	5.92 (0.38)	1.06 (0.24)	5.27 (0.33)	0.92 (0.23)	**5.43 (0.35)**	**0.85 (0.22)**

The parameter estimates from the best-fitting model for each age group are highlighted in bold.

## Supplementary information

Supplemental Material

## Data Availability

Data will be made available on Open Science Framework (https://osf.io/mjy8w/) upon publication.

## Code availability

Code used to produce the figures and analyses will be made available on Open Science Framework (https://osf.io/mjy8w/) upon publication.
