# Supplementary material for: The rational use of causal inference to guide reinforcement learning strengthens with age
Source: NPJ Sci Learn. 2020 Oct 27;5:16. doi: 10.1038/s41539-020-00075-3 (PMC7591882; doi:10.1038/s41539-020-00075-3)
Supplement: Supplementary file 1 — Supplemental Material [file 41539_2020_75_MOESM1_ESM.pdf]

Supplementary Information

Supplemental Behavioral Analyses

To assess how participant's attributions changed over the course of the task, we added z-scored trial number as a covariate of no interest to the analysis of the attributions. We found that the proportion of attributions decreases across the task ($\chi^2(1, N = 90) = 7.13$, $p = 0.008$; Supplementary Figure 1). The addition of this covariate did not change the pattern of results reported in the main text (see Supplementary Table 1 for full statistics from the model).

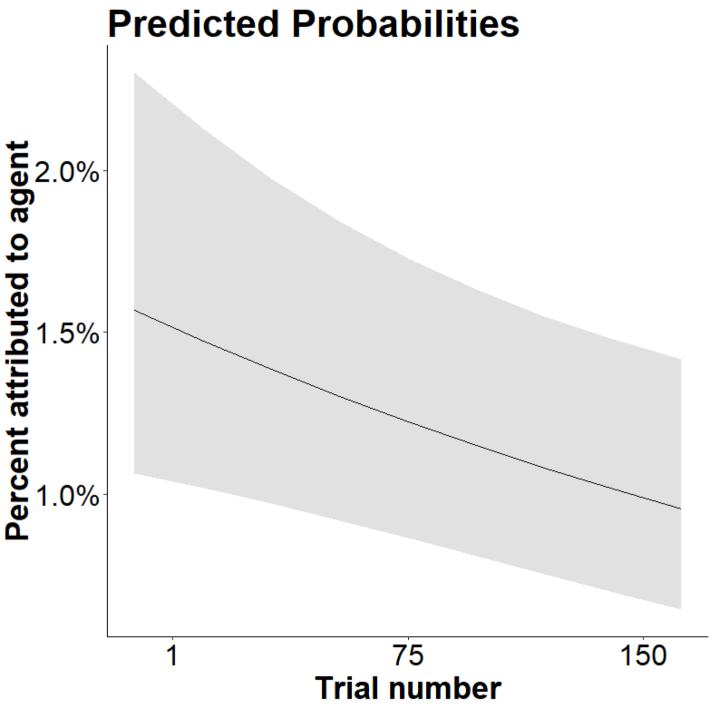

Supplementary Figure 1. Predicted probability of proportion of trials attributed to hidden agents decreases across trials.

Supplementary Table 1. Summary of statistics from attribution model including trial number as a covariate of no interest

	χ^2	DF	p-value
(Intercept)	65.44	1	< 0.001
Reward Outcome	7.80	1	0.0052
Territory	45.34	2	< 0.001
Age	16.87	1	< 0.001
Age^2	6.04	1	0.014
Trial number	7.13	1	0.0076
Reward Outcome * Territory	86.36	2	< 0.001
Reward Outcome * Age	4.90	1	0.027
Reward Outcome * Age^2	0.78	1	0.38
Territory * Age	2.29	2	0.32
Territory * Age^2	4.76	2	0.093
Reward Outcome * Territory * Age	0.17	2	0.92
Reward Outcome * Territory * Age^2	0.19	2	0.91

Modeling the interactions between trial number and the other fixed effects led to the mixed effects model failing to converge, indicating that we cannot interpret the effects from this model and that we likely do not have sufficient data to support this analysis without removing random or fixed effects (Bates et al., 2015). While we were not able to assess if changes in attribution over time varied by territory, reward outcome, or age, the results indicate that with time, as performance on the task improves, attribution rates become lower.

To address the possibility of territory presentation order effects, we ran two variants of the learning analysis reported in the main text: one including the presentation order as a covariate of no interest and one including the first territory presented as a covariate of no interest. The models indicated no significant effect of presentation order ($\chi^2(5, N = 90) = 1.24, p = 0.94$) or which

territory was presented first ($\chi^2(2, N = 90) = 0.56, p = 0.76$) on learning. The addition of these covariates did not change the pattern of results reported in the main text (see Supplementary Tables 2 and 3 for full statistics from the model).

Supplementary Table 2. Summary of statistics from learning model including presentation order as a covariate of no interest

	χ^2	DF	p-value
(Intercept)	242.36	1	< 0.001
Trial number	100.47	1	< 0.001
Territory	3.15	2	0.21
Age	13.27	1	0.00027
Age^2	5.26	1	0.022
Presentation order	1.24	5	0.94
Trial number * Territory	15.34	2	0.00047
Trial number * Age	3.80	1	0.051
Trial number * Age^2	1.91	1	0.17
Territory * Age	0.49	2	0.78
Territory * Age^2	6.93	2	0.031
Trial number * Territory * Age	1.89	2	0.39
Trial number * Territory * Age^2	6.83	2	0.033

Supplementary Table 3. Summary of statistics from learning model including first territory as a covariate of no interest

	χ^2	DF	p-value
(Intercept)	245.99	1	< 0.001
Trial number	100.50	1	< 0.001
Territory	3.21	2	0.20
Age	14.55	1	0.00014
Age^2	5.30	1	0.021
First territory	0.56	2	0.76
Trial number * Territory	15.47	2	0.00044
Trial number * Age	3.80	1	0.051
Trial number * Age^2	1.91	1	0.17
Territory * Age	0.45	2	0.80
Territory * Age^2	6.88	2	0.032
Trial number * Territory * Age	1.91	2	0.38
Trial number * Territory * Age^2	6.80	2	0.033

To determine the extent to which learning generalizes across environments and how this might be influenced by which territory was encountered first, we added an interaction between learning block number and first territory to the analysis of the learning data. This led to the mixed effects model failing to converge, indicating that we cannot interpret the effects from this model and that we likely do not have sufficient data to support this analysis without removing random or fixed effects (Bates et al., 2015). However, including learning block and first territory as covariates in the learning analysis allowed the model to converge. While we still did not find a significant effect of which territory was presented first ($\chi^2(2, N = 90) = 0.55$, $p = 0.76$) on learning, there was a significant effect of learning block ($\chi^2(2, N = 90) = 6.91$, $p = 0.032$; see Supplementary Table 4 for full statistics from the model). This effect appeared to be driven by higher percent optimal

choice in the third block relative to the first two blocks (see Supplementary Figure 2 below). Modeling the interactions between block and the other covariates of interest also led to a convergence error, precluding us from examining any possible age by block interactions. This result provides some evidence for a benefit for generalization of learning by the third block of the task.

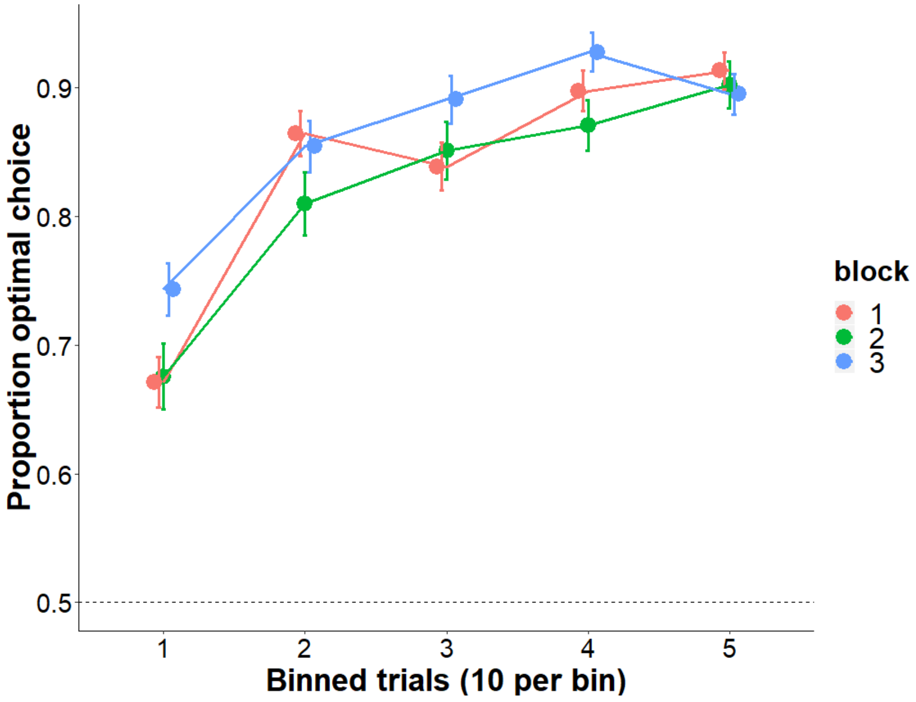

Supplementary Figure 2. Better performance overall by the third block of the task relative to the first two blocks, across ages.

Supplementary Table 4. Summary of statistics from learning model including first territory and block number as a covariate of no interest

	χ^2	DF	p-value
(Intercept)	246.10	1	< 0.001
Trial number	100.50	1	< 0.001
Territory	3.97	2	0.14
Age	14.62	1	0.00013
Age^2	5.42	1	0.020
First territory	0.55	2	0.76
Learning block	6.91	2	0.032
Trial number * Territory	15.53	2	0.00043
Trial number * Age	3.88	1	0.049
Trial number * Age^2	1.93	1	0.16
Territory * Age	1.03	2	0.60
Territory * Age^2	6.74	2	0.034
Trial number * Territory * Age	1.83	2	0.40
Trial number * Territory * Age^2	6.71	2	0.035

Finally, we examined reaction times during the learning phase by running an analysis of the choice reactions times that paralleled the analysis of optimal choice reported in the main text, including trial-wise optimal choice by territory, trial number within a territory, continuous age, continuous age-squared and their interactions. This analysis revealed that reaction time decreased across the task ($\chi^2(1, N = 90) = 5.85, p = 0.015$) and decreased with age ($\chi^2(1, N = 90) = 37.42, p < 0.001$), indicating that reaction time became faster across the task and with increasing age (Supplementary Figure 3). There was also a significant main effect of age-squared ($\chi^2(1, N = 90) = 12.56, p = 0.00040$). There were no significant interactions between trial number

and age (χ^2(1, N = 90) = 0.24, p = 0.62) or age squared (χ^2(1, N = 90) = 0.086, p = 0.77), indicating that reaction time generally decreased across the task and across all ages. The were no statistically significant effects of territory, territory by trial number, territory by age, territory by age-squared, trial number by territory by age, or trial number by territory by age-squared on reaction time (all χ^2s < 1.2, *p*s > 0.56).

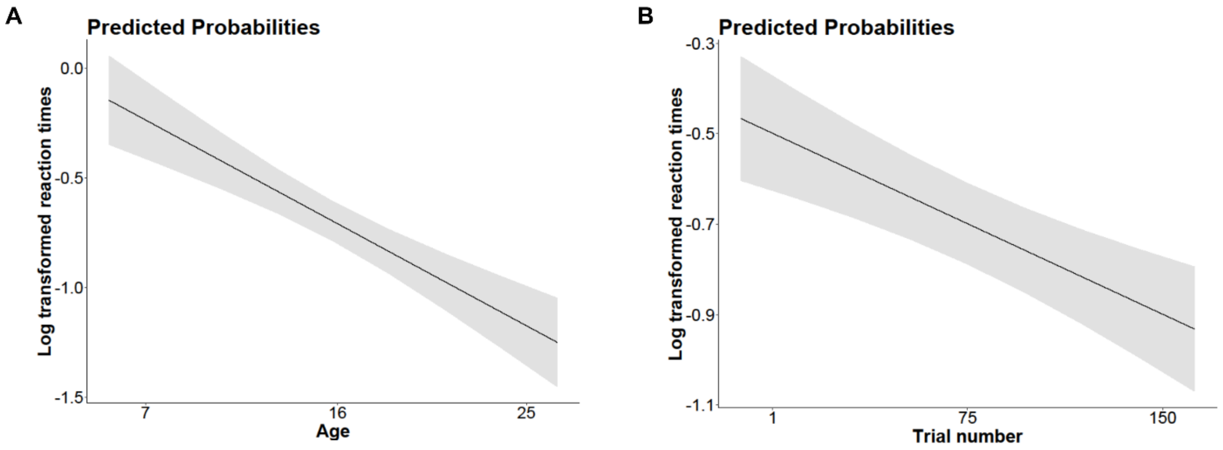

Supplementary Figure 3. Predicted probabilities of log transformed reaction times decrease across age and trial number.

Model Fitting

All reinforcement learning models were fit using mfit, an openly available repository which finds the maximum a posteriori estimates of the parameters for each subject (https://github.com/sjgershm/mfit). Uniform priors were assumed for all parameters except for the inverse temperature parameter, for which prior distribution was β ~ Gamma(4.82, 0.88), based on previous research (Dorfman et al., 2019; Gershman, 2016). The same priors were used for all age groups. See Supplementary Table 4 for the complete reporting of protected exceedance probabilities (PXPs) for each model by age group.

Supplementary Table 3. Summary of PXP values from model comparison results

	One Learning Rate	Two Learning Rate	Three Learning Rates (one per territory)	Empirical Bayesian by Territory	Adaptive Bayesian	Noisy Bayesian	Empirical Bayesian
Children	0.9817	0.0013	0.0099	0.0008	0.0047	0.0008	0.0009
Adolescents	0.0986	0.0032	0.0028	0.0028	0.8858	0.0028	0.0040
Adults	0.0548	0.0773	0.0268	0.0270	0.0394	0.0268	0.7479

Empirical Bayesian Reinforcement Learning Model

The model assumes that participants choose an option (c) with an intrinsic reward distribution. The reward distribution can be altered by latent agent intervention $(z = 1)$. The marginal distribution over reward given the chosen option is:

$$P(r|c) = P(r|c, z = 0)P(z = 0) + P(r|z = 1)P(z = 1)$$

where the participant obtains a reward (r) from the intrinsic distribution $P(r|c, z = 0)$ with probability $P(z = 0)$ or receives a reward from the distribution altered by the latent agent $P(r|z = 1)$ with probability $P(z = 1)$. $P(z = 1)$ is derived by averaging each participant's explicit attributions over the course of the experiment.

Rewards in the task are binary (gold or rocks) and each option is selected from a Bernoulli distribution Bern(θ^c). When there is no latent agent intervention, the probability of obtaining a reward of 1 (gold) is θ^c and the probability of obtaining a reward of 0 (rocks) is 1 - θ^c. Regardless of the participant's choice, the Millionaire latent agent intervention yields reward ($P(r = 1|z = 1) = 1$), the Robber latent agent intervention yields no reward ($P(r = 0|z = 1) = 1$), and the Sheriff latent agent intervention yields a 0.5 probability of reward ($P(r = 1|z = 1) = 0.5$). The participant is not aware of the reward probabilities associated with

the options and uses experienced outcomes to update her prior belief that parameters θ^c are independently distributed according to $Beta(a,b)$, where a and b are both equal to 1 (a uniform distribution).

The participant uses a Bayesian update rule that takes the form of a reinforcement learning update rule that incorporates latent agent inference to update her estimate of the θ^c reward probability:

$$\theta_{t+1} = \theta_t + \alpha_t(r_t - \theta_t)$$

where the reward prediction error is scaled by the learning rate α_t:

$$\alpha_t = \frac{P(z_t = 0|r_t, c_t)}{N_t^c + a + b}$$

The learning rate is based on the extent to which the participant believes that a trial's outcome is due to her own actions. The denominator represents the accumulated evidence up to trial *t* about the intrinsic reward probability, with N_t^c representing the sum of past beliefs about lack of latent agent intervention for a given choice option. The numerator represents the posterior probability of the participant's belief about latent agent intervention given the feedback for the present choice. $P(z = 0|r, c)$ is stipulated by Bayes' rule:

$$P(z|r,c) = \frac{P(r|z,c)P(z)}{\sum_{z'} P(r|z',c)P(z')}$$

This leads to different expressions of the posterior probability of latent agent intervention that depend on the latent agent and the feedback, where the probability of intervention $P(z = 1)$ is known and the participant's inference about latent agent intervention depends her estimate of θ^c:

Millionaire/Negative Feedback: $P(z = 0|r = 0, c) = 1$

Millionaire/Positive Feedback: $$P(z = 0|r = 1, c) = \frac{\theta^c P(z = 0)}{\theta^c P(z = 0) + P(z = 1)}$$

Robber/Negative Feedback: $$P(z = 0|r = 0, c) = \frac{(1 - \theta^c)P(z = 0)}{(1 - \theta^c)P(z = 0) + P(z = 1)}$$

Robber/Positive Feedback: $P(z=0|r=1,c)=1$

Sheriff/Negative Feedback: $P(z=0|r=0,c)=\dfrac{(1-\theta^c)P(z=0)}{(1-\theta^c)P(z=0)+P(z=1)/2}$

Sheriff/Positive Feedback: $P(z=0|r=1,c)=\dfrac{\theta^c P(z=0)}{\theta^c P(z=0)+P(z=1)/2}$

Since learning rates in this model vary based on the participant's estimate that the agent did not intervene, intuitively the learning rate is larger when the outcome cannot be attributed to the latent agent and is smaller when it can be attributed to the latent agent. Thus, lower learning rates reflect outcomes that yield less information about the intrinsic reward probability associated with an action.

Adaptive Bayesian Reinforcement Learning Model

The participant's estimate of latent agent intervention probability on each trial $P(z_t=1)$ can be represented by the variable ω. The ω update rule is approximated by the average of the participant's past estimates of latent agent intervention such that:

$$\omega_{t+1}=\omega_t+\frac{1}{t+a+b}(P(z_t=1|r_t,c_t)-\omega_t)$$

The ω update rule replaces $P(z=1)$ in the Bayesian update rule described above.

Model fits by age

To gain a better understanding of the distribution of best fitting models across continuous age, we examined the posterior probability that each model generated the data for each subject. We determined the best fitting model for each subject by selecting the model with the highest posterior probability and plotted the best fitting model by age with subjects fit as a single group (Supplementary Figure 4) and with subjects fit by the age groups reported in the main text (Supplementary Figure 5). Bayesian models are depicted in shades of red and non-Bayesian reinforcement learning models are depicted in shade of blue. Additionally, we have also plotted

the proportion of best fitting models based on BIC values by age group and model (Supplementary Figure 6). Consistent with the PXP results, though we observed some heterogeneity in the best fitting models across age, the one learning rate, adaptive Bayesian, and empirical Bayesian models were the most prominent best-fitting models across all metrics. In the plots examining posterior probabilities (Supplementary Figures 4 and 5), the general distributions of Bayesian versus non-Bayesian model fits are similar regardless of whether subjects were fit as a single group or by age group, with some individual differences in which model fits certain subjects best. Together, these results suggest that whereas adults and adolescents tend to show comparable learning that incorporates beliefs about the causal structure of the environment, children did not tend to similarly use the structure of the environment to guide learning.

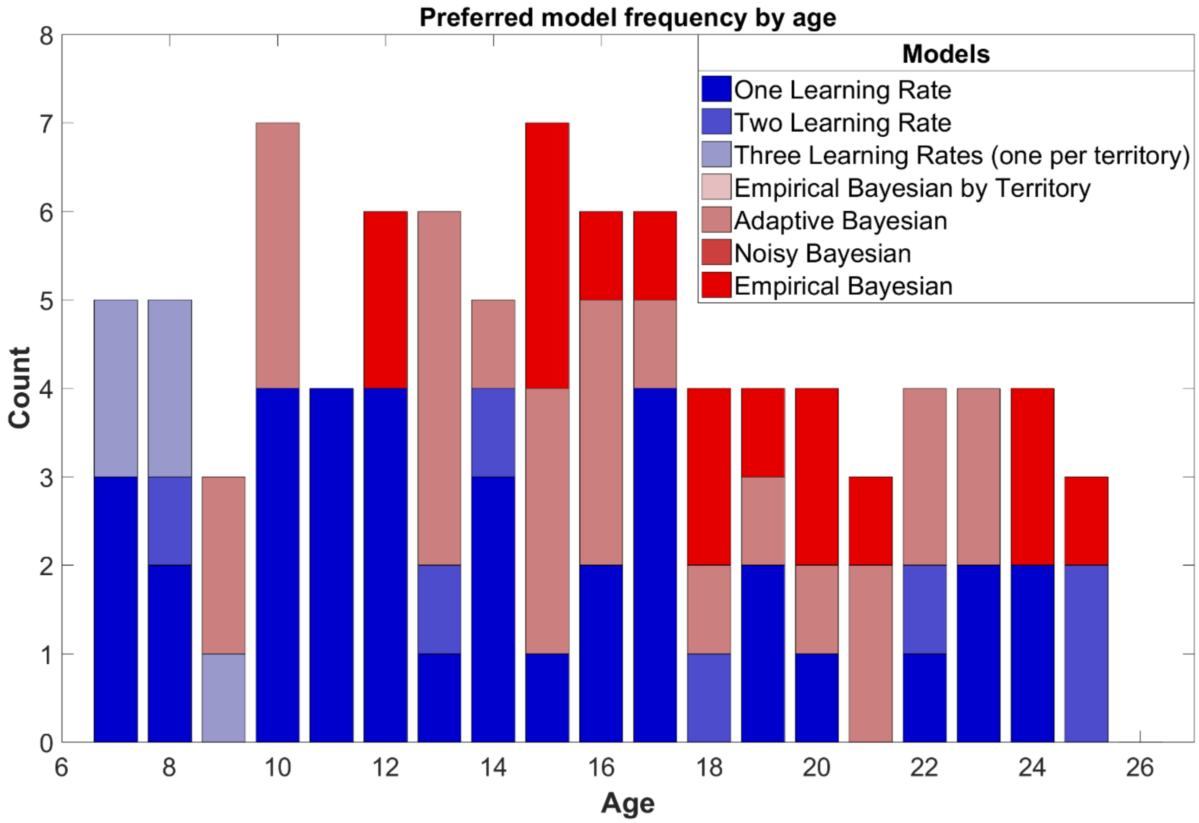

Supplementary Figure 4. The distribution of best fitting models when subjects were fit as a single group (determined by selecting the highest subject-specific posterior probability amongst the seven models) by continuous age shows some heterogeneity in best-fitting models across participants.

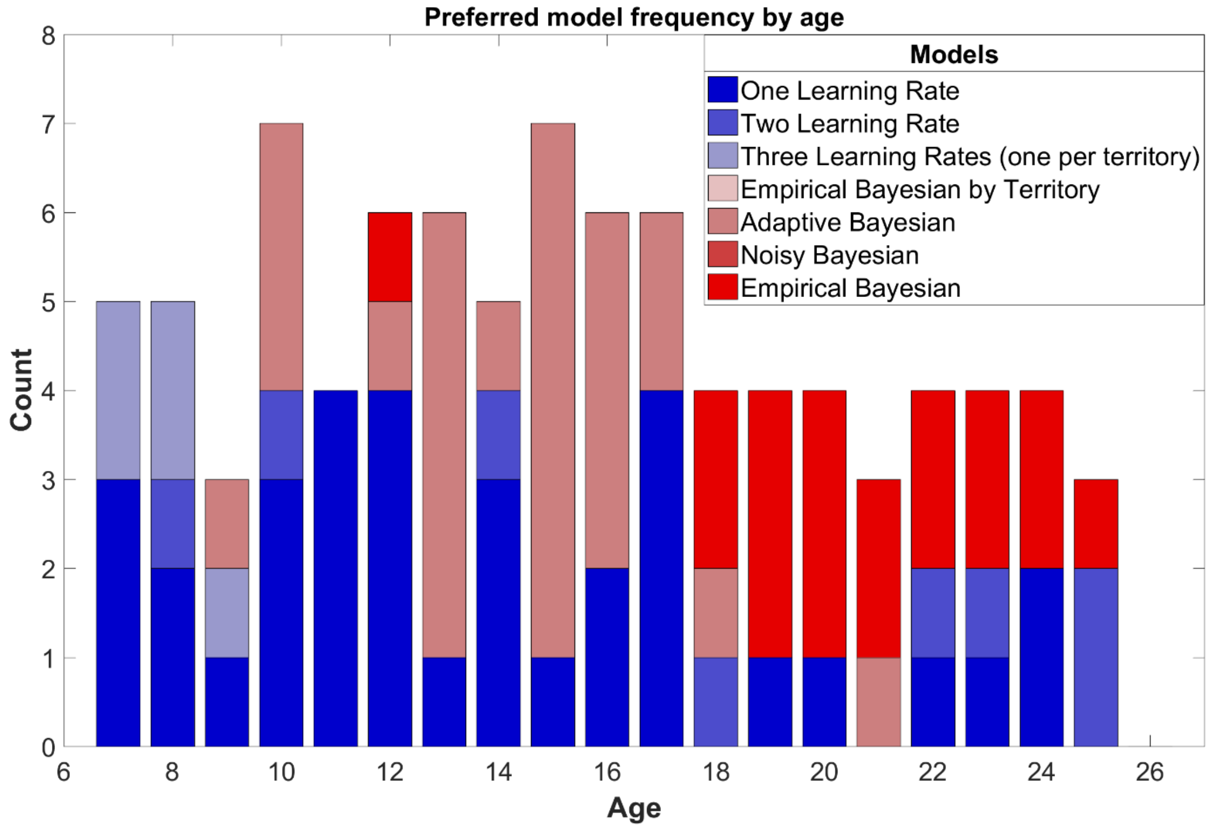

Supplementary Figure 5. The distribution of best fitting models when subjects were fit as by age groups (determined by selecting the highest subject-specific posterior probability amongst the seven models) by continuous age shows some heterogeneity in best-fitting models across participants.

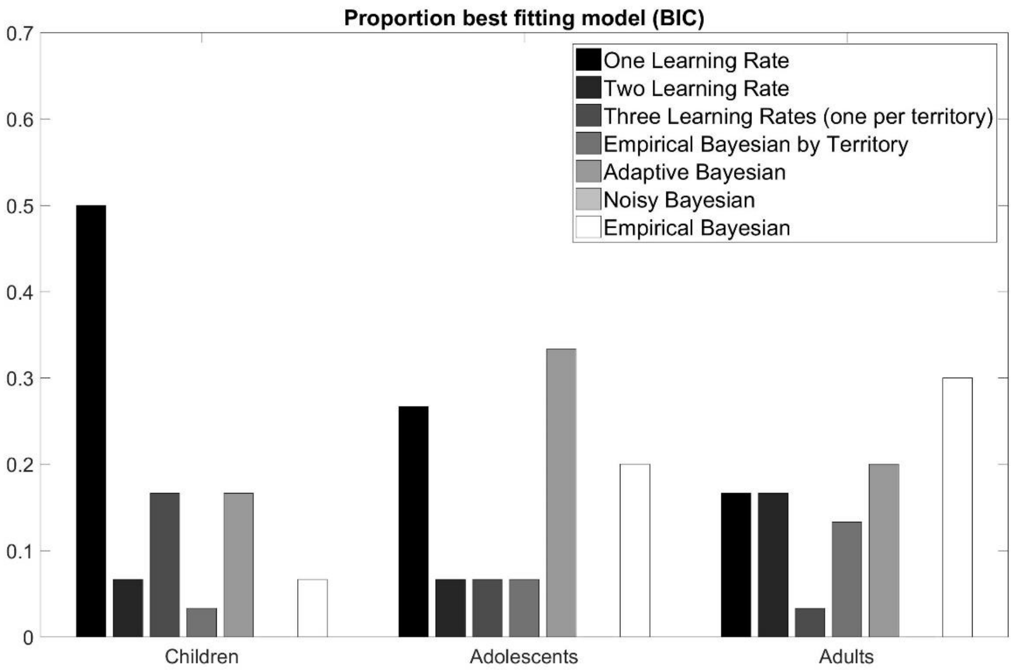

Supplementary Figure 6. The distribution of best-fitting models by age group based on BIC values.

Fitted parameter distributions by age group

Fitted parameter distributions for each age group and each of the best fitting models are included below to visualize differences: the one learning rate (Supplementary Figure 7), adaptive Bayesian (Supplementary Figure 8), and empirical Bayesian (Supplementary Figure 9).

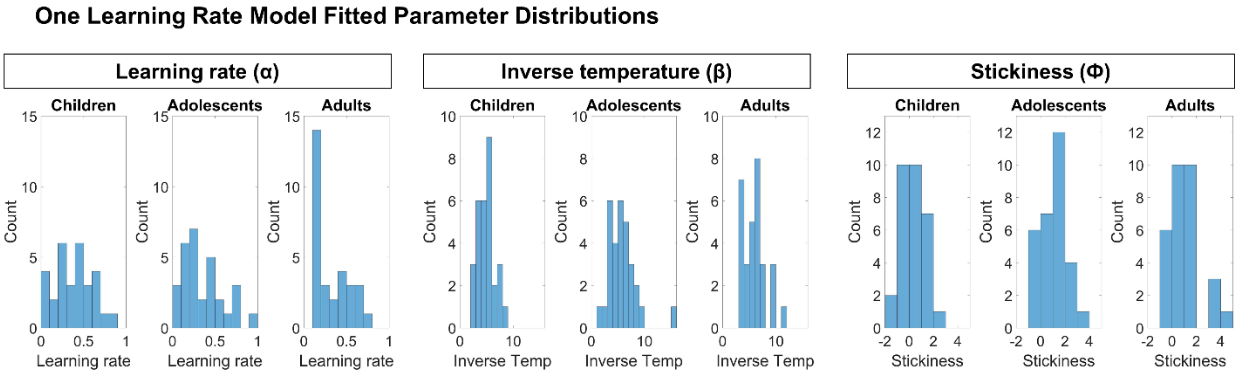

Supplementary Figure 7. The distribution of fitted parameters from the one learning rate model.

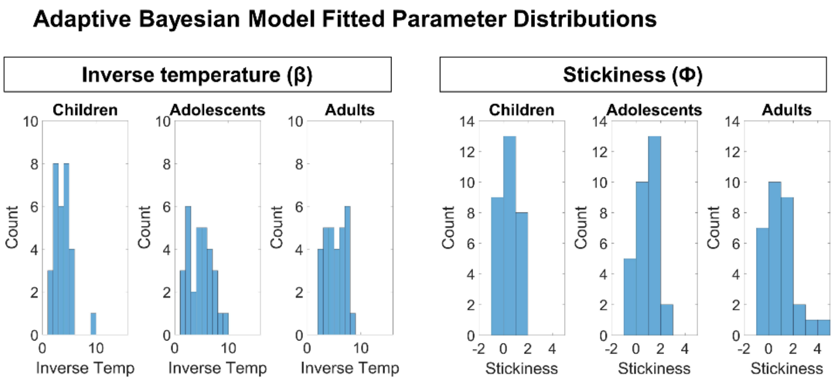

Supplementary Figure 8. The distribution of fitted parameters from the adaptive Bayesian rate model.

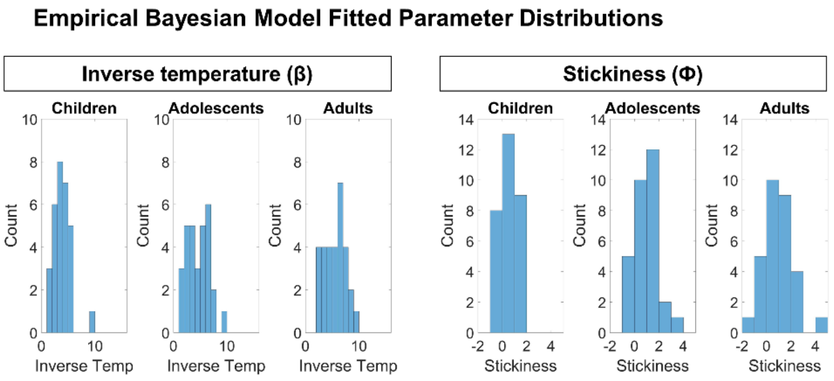

Supplementary Figure 9. The distribution of fitted parameters from the adaptive Bayesian rate model.
